# The Impact of COVID-19 Vaccination Side-Effects on Work Attendance among Saudi Healthcare Workers

**DOI:** 10.3390/idr16040059

**Published:** 2024-08-19

**Authors:** Jawaher Alguraini, Mohamed T. S. Saleem, Nahed N. Mahrous, Abbas Shamsan, Fatima Zia Zaidi, Ohoud S. Alhumaidan, Yahya F. Jamous

**Affiliations:** 1 Department of Research Center, Dr. Sulaiman Al Habib Medical Group, Riyadh 12214, Saudi Arabia; jawaherhamad18@gmail.com (J.A.); abbas.shamsan@drsulaimanalhabib.com (A.S.); 2 College of Pharmacy, Nursing and Medical Sciences, Riyadh ELM University, Riyadh 13244, Saudi Arabia; tsmohamed.saleem@riyadh.edu.sa; 3 Department of Biological Sciences, College of Science, University of Hafr Al-Batin, Hafr Al-Batin 39524, Saudi Arabia; nnmahrous@uhb.edu.sa; 4 Department of Academic Affairs, Dr. Sulaiman Al Habib Medical Group, Riyadh 12214, Saudi Arabia; fatimaziazaidi@gmail.com; 5 Clinical Laboratory Sciences Department, College of Applied Medical Sciences, King Saud University, Riyadh 12372, Saudi Arabia; oalhumaidan@ksu.edu.sa; 6 Vaccine and Bioprocessing Centre, King Abdulaziz City for Science and Technology (KACST), Riyadh 11442, Saudi Arabia

**Keywords:** COVID-19, SARS-CoV-2, vaccines, healthcare workers, side-effects, work absenteeism, mRNA vaccine, viral vector vaccine, inactivated virus vaccine, comorbidities

## Abstract

Objective: This cross-sectional-survey-based study aimed to investigate the severity of side-effects from Coronavirus disease (COVID-19) mRNA (Pfizer, Moderna), viral vector DNA (Oxford-AstraZeneca, J&J/Janssen), inactivated virus (Sinopharm, Sinovac), and other vaccines among healthcare workers (HCWs) in Saudi Arabia, focusing on their impact on work attendance. Methods: A total of 894 HCWs residing in Saudi Arabia participated in this study from March 2023 to May 2023. Participants completed an online questionnaire assessing demographic information, vaccination status, comorbidities, vaccine side-effects, and missed work information after vaccination. Descriptive statistics and chi-square tests were used for data analysis. Results: The majority of participants were female (83.7%) and aged 25–34 years (42.8%). Most participants were predominantly vaccinated with mRNA vaccines. Common side-effects included pain at the injection site, fatigue, fever, and chills. However, no significant association was found between vaccine type, side-effects, and work absenteeism. While demographic factors such as age and healthcare profession did not influence work absenteeism, variations were observed among different racial groups. Conclusion: COVID-19 vaccination among HCWs in Saudi Arabia is associated with common side-effects, but their impact on work attendance is not significant. Understanding these implications can inform strategies to support the healthcare workforce and mitigate the impact on patient care and staffing during the ongoing COVID-19 pandemic.

## 1. Introduction

Coronavirus disease (COVID-19), caused by severe acute respiratory syndrome coronavirus 2 (SARS-CoV-2), was first reported in Wuhan, China, in December 2019, with the virus spreading globally rapidly [1]. The causative virus, SARS-CoV-2, is a single-stranded RNA virus within the family coronaviridae that affects the respiratory tract system and other organs of the body [2]. In March 2020, the World Health Organization (WHO) declared COVID-19 as a pandemic because of its fast transmission among humans and deadly SARS-CoV-2 cause [1]. Severe acute respiratory syndrome coronavirus 2 is a highly contagious virus that is transmitted through respiratory droplets, direct human contact, and fomites with variable degrees of pathogenicity. The pervasive presence of COVID-19 has impacted every nation and geographical location of the world, sparing none from its far-reaching consequences.

Globally, the COVID-19 pandemic has claimed millions of lives since its outbreak in early 2020 [3]. Consequently, health authorities had set strict rules and precautions to conduct risk assessments and contain the outbreak as it was not clear when effective vaccines may be available. Such restrictive measures depend on various biological, environmental, and social factors to prevent transmission. These included, but were not limited to, the closure of cultural events, the prohibition of social events and gatherings, the confinement of individuals to their homes, and lockdowns to limit infection transmission (e.g., social distancing, frequent hand washing and sanitizing) and ultimately contain the COVID-19 outbreak. Educational institutions were closed, and home-based learning via online teaching was among these measures, resulting in a decrease in the spread of COVID-19 and a more sedentary lifestyle [4,5].

Challenges with the pandemic crisis continued in developing effective vaccines as well as validating their efficacy and adverse reactivity towards the novel SARS-CoV-2. Extraordinarily, several vaccines had been developed globally within one year and had undergone various evaluation stages as of July 2020 [6,7]. These vaccines build upon various platforms including virus-vectored vaccines, protein subunit vaccines, genetic vaccines, and monoclonal antibodies, although most vaccines were developed using the S-protein of SARS-CoV-2 [7,8]. In December 2020, several vaccines were launched after being authorized for emergency use in many countries to overcome the COVID-19 pandemic [9]. Vaccines mRNA-BNT162b2 (BioNTech and Pfizer) and mRNA-1273 (Moderna) COVID-19 were the first authorized as a course of two doses by the Food and Drug Administration (FDA) and European Medicines Agency (EMA), respectively, in many countries including the United Kingdom, Bahrain, Canada, Mexico, Saudi Arabia, the USA, Switzerland, EUA, and Japan [10,11]. In addition, ChAdOx1 nCoV-19 (AstraZeneca/Oxford) and Ad26.CoV2-S (Johnson & Johnson; Janssen) are adenovirus-vectored vaccines developed towards SARS-CoV-2 and were approved for emergency use on December 2020. AstraZeneca was only accepted by the UK regulatory authority Medicines and Healthcare Regulatory Agency (MHRA) for emergency use at first in Europe, while the Janssen vaccine was approved by the US FDA. Early in 2021, both vaccines were postponed as they resulted in rare thrombocytopenia and thrombosis side-effects after a single-dose administration [9].

Nonetheless, infectious disease outbreaks, such as the COVID-19 pandemic, exert a detrimental effect on the physical, social, and psychological aspects of both individuals and societies, resulting in consequential and notable economic implications [11,12]. On March 2020, the first COVID-19 case was officially recorded in Saudi Arabia, coinciding with the global trend of escalating case numbers worldwide [13]. In response to the outbreak, the Saudi Ministry of Health implemented a lockdown and enforced a curfew for all its residents by the end of March 2020 which was upheld for approximately three months [14]. The impact of the virus was felt on various fronts, including a significant impact on psychological well-being, general physical health, and the environment [15,16]. The measures implemented during the COVID-19 lockdown period influenced various facets of human behaviours and activities, encompassing vehicle utilization, public transit systems, and industrial operations [17].

The safety and the immunogenicity of COVID-19 vaccines have been evaluated in individuals along with the duration of protection against COVID-19 provided by the vaccine. Healthcare workers, the elderly, and patients with chronic diseases are critical populations that should be vaccinated early. The success of any vaccination programme depends on high vaccine hesitancy. A research investigation conducted among healthcare workers (HCWs) in China revealed a substantially greater inclination towards accepting COVID-19 vaccination in comparison to the broader population [18]. However, there is a lack of comprehensive data pertaining to the adverse repercussions resulting from side-effects associated with vaccines in the healthcare sector. This knowledge gap can be attributed to the infrequent and mild nature of adverse events related to established vaccines, such as those for influenza, which typically results in side effects that last for brief periods and typically do not significant affect work performance [19].

In contrast, the short-term side-effects associated with the currently available COVID-19 mRNA vaccines are more frequent and may lead to great discomfort with a greater impact on work. Initial safety data for both mRNA vaccines indicated that systemic symptoms, such as fatigue and headaches, were reported by over 50% of individuals who received a second vaccination dose [20,21]. On the 21st of June 2020, the nationwide lockdown which lasted for 73 days in Saudi Arabia ended, allowing residents of the Kingdom to return to normal life [13]. In light of the fact that social distancing was effective in limiting COVID-19 transmission to a minimum, achieving herd immunity was contingent upon a successful and widespread vaccination programme. In Saudi Arabia, the Ministry of Health launched a vaccination campaign using the “Sehaty” mobile application, which was designed to streamline the COVID-19 vaccine registration process. Additionally, vaccination centres were established in various urban areas across the nation [22]. HCWs are critical in the fight against COVID-19. Ensuring their availability and attendance at work is vital for maintaining healthcare services. However, vaccine-related side-effects can impact their ability to attend work. This study aims to investigate the severity of side-effects from various COVID-19 vaccines among HCWs in Saudi Arabia and their effect on work attendance.

## 2. Method

### 2.1. Ethical Considerations

Ethical clearance was obtained from the relevant institutional review board (IRB) prior to data collection, under the ethical code RC22.10.03, with the approval date of 13 October 2022. Informed consent was obtained from all participants before their inclusion in this study, emphasizing voluntary participation and the confidentiality of their responses.

### 2.2. Study Design

A cross-sectional-survey-based study was conducted from March 2023 to May 2023 among HCWs living all over the Kingdom of Saudi Arabia. Self-reported online questionnaires were chosen as the data collection method in order to maximize outreach within a short span of time. The primary aim of this study was to investigate the severity of side-effects from COVID-19 mRNA (Pfizer Inc., New York, NY, USA; Moderna, Inc., Cambridge, MA, USA), viral vector DNA (Oxford-AstraZeneca, Oxford, UK; Johnson & Johnson/Janssen, New Brunswick, NJ, USA), inactivated virus (Sinopharm, Beijing, China; Sinovac, Beijing, China), and any other vaccines mentioned in the questionnaire among Saudi HCWs, focusing on whether these side-effects are severe enough to cause missed work.

### 2.3. Participant Recruitment

Participants were recruited through various channels, including healthcare facilities, professional networks, and social media platforms. Recruitment materials were disseminated in Arabic and English languages to ensure accessibility for all eligible participants.

### 2.4. Inclusion Criteria

All healthcare workers aged 18 years and above, residing in Saudi Arabia, and who had received at least one dose of a COVID-19 vaccine were eligible for participation. Participants providing incomplete information were excluded from this study.

### 2.5. Sample Size Determination

The sample size was determined using the single population proportion formula. With a probability of missing work post vaccination set at 50%, a margin of error of 5%, and a confidence level of 95%, the required minimum sample size was calculated to be 385. However, to increase the power of this study, the sample size was increased to 894 participants.

### 2.6. Data Collection

A structured questionnaire comprising 20 multiple-choice questions was developed to collect relevant information from participants. The questionnaire covered demographic details, vaccination status, comorbidities, vaccine side-effects, and missed work information after vaccination. Participants were asked to specify the type of vaccine received for each dose (first, second, and third), including mRNA (Pfizer Inc., New York, NY, USA; Moderna, Inc., Cambridge, MA, USA), viral vector DNA (Oxford-AstraZeneca, Oxford, UK; Johnson & Johnson/Janssen, New Brunswick, NJ, USA), inactivated virus (Sinopharm, Beijing, China; Sinovac, Beijing, China) and other types of vaccines. The questionnaire was made available online through Google Forms and distributed via social media platforms, ensuring ease of access and participation.

### 2.7. Study Outcome

The primary outcome of this study was the severity of side-effects experienced by HCWs following each dose of the COVID-19 vaccine, with a focus on whether these side-effects led to missed work. Secondary outcomes included factors associated with side-effects and missed work after each stage of vaccination.

### 2.8. Statistical Analysis

Descriptive statistics, such as frequency and percentage, were used to summarize participant characteristics and the prevalence of side effects. The chi-square test was employed to assess associations between categorical variables, with statistical significance set at *p* < 0.05. Data analysis was performed using the Statistical Package for the Social Sciences (SPSS) version 26 (IBM Corp., Armonk, NY, USA). 

## 3. Results

**Figure 1:**
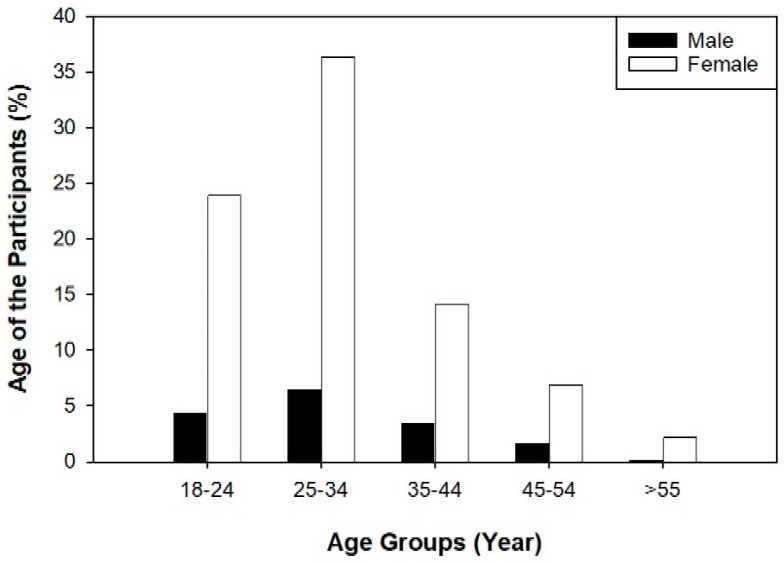
Age of the healthcare participants in this study. Most participants were among 25 to 34 years. Female participants represent the majority in total.

The total number of participants in this study was 894, with the majority falling within the age group of 25–34, accounting for 42.8% of the total population. The mean age of the participants was 30.6 ± 9.2 (Figure 1). Female participants constituted the majority at 83.7%, while male participants accounted for 16.3% (Table 1). From the entire sample pool, in terms of racial groups, the greatest number comprised the Asian population, accounting for 56.3% of the enrolment, while the remaining percentage was a mix of racial groups (Arabs, African, or White). In terms of the geographical location where participants were from, the majority were from the middle region of the country (64.4%). Furthermore, 86.6% of the population reported that they were non-smokers, with 69.7% of the population reporting exercising occasionally in their life. The percentage of participants reporting co-morbidities was 74.7%. Asthma (9.7%) and hypertension (6%) were observed in some participants compared to other co-morbidities. Most of the population reported no surgical history. In terms of healthcare profession, 72.4% of the participants were from the nursing category. The results indicated that all the 894 participants received one dose of the COVID-19 vaccine and, finally, out of 894 participants, 79% of the population reported being fully vaccinated with three doses of the COVID-19 vaccine (Table 1).

**Figure 2:**
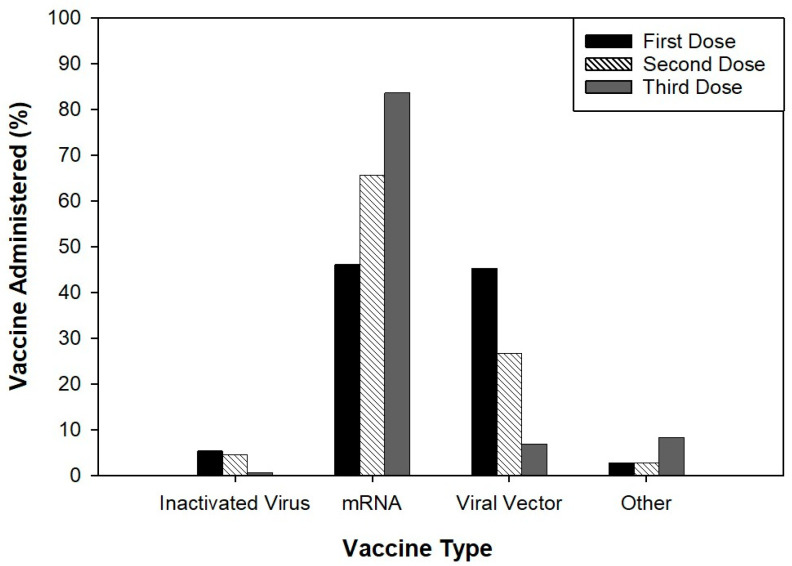
Type of vaccine administered including inactivated virus, mRNA, viral vector, and other vaccines.

**Figure 3:**
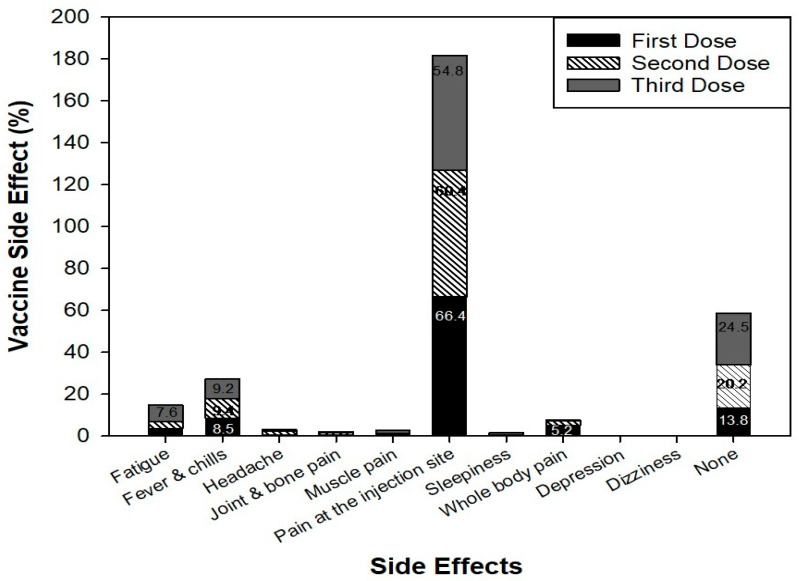
Reported vaccine side-effects during the first, second, and third doses of the vaccine.

Among the 894 participants, 413 of them received the mRNA vaccine for their first dose (46.2%) and 406 participants received the viral vector vaccine (45.4%). For the second vaccination dose, 587 participants received the mRNA vaccine, and for the third dose, 748 of the participants received the mRNA vaccine (Figure 2). Most participants reported pain at the injection site as a side-effect for the first (594), second (540), and third (490) doses of vaccines. Other side-effects reported included fatigue, fever, and chills (Figure 3).

**Figure 4:**
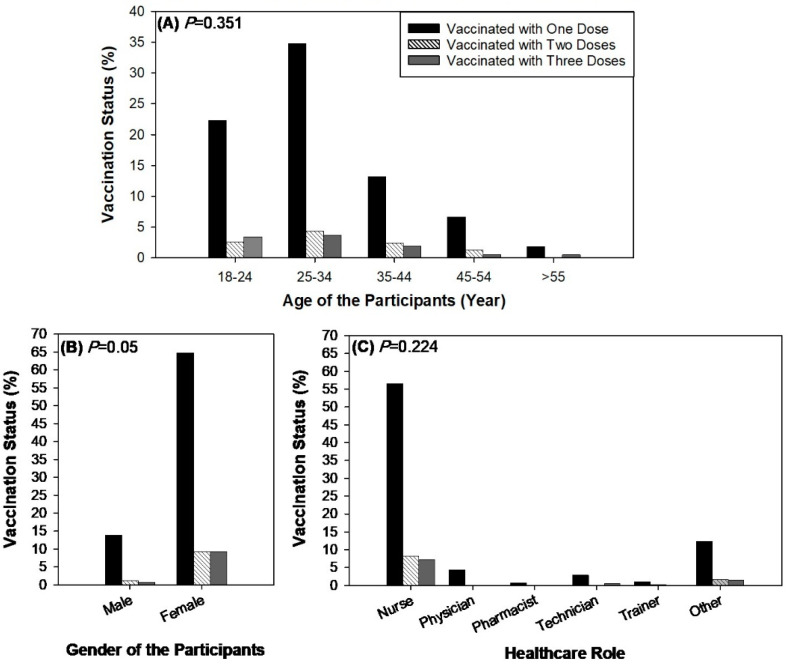
Association between status of vaccine and demographic variables of the participants, (**A**) age, (**B**) gender, and (**C**) healthcare role, during the first, second, and third doses of the vaccine.

**Figure 5:**
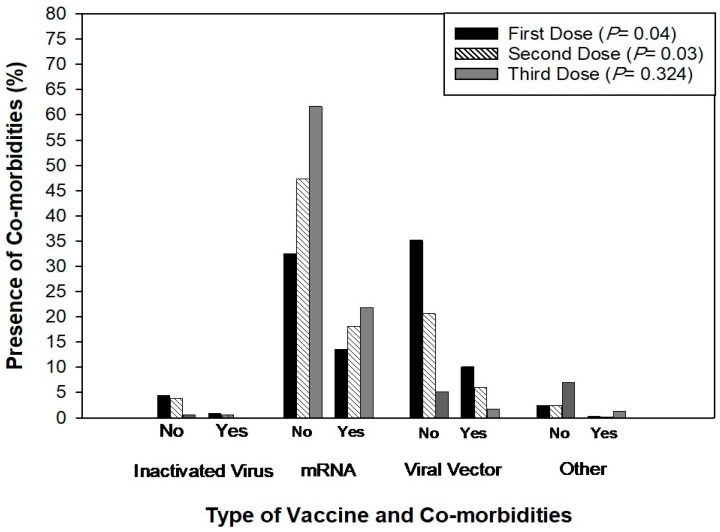
Association between the presence of co-morbidities and vaccine type during the first, second, and third doses of the vaccine.

**Figure 6:**
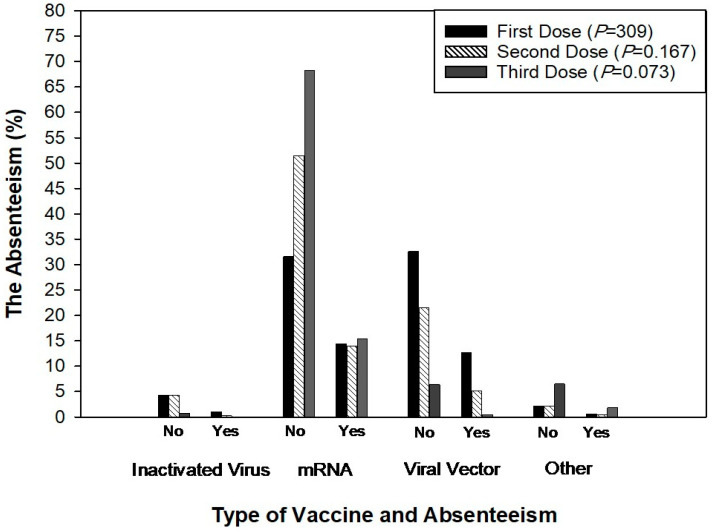
Association between vaccine type and the need for work absence after the first, second, and third vaccine doses.

There was a significant (*p* < 0.05) association between gender and vaccination status; however, the association between age, healthcare role, and vaccination status was found to be insignificant (Figure 4). Furthermore, there was a significant association between the type of vaccine and the presence of comorbidities during the first (*p* < 0.04) and second vaccination doses (*p* < 0.03) (Figure 5). There was no significant association between the type of vaccine and work absence during the first, second, and third doses of vaccination (Figure 6). Similarly, there was no significant association between side-effects and the need to be absent from work during the first, second, and third doses of vaccination (Table 2). Based on the results, it is observed that out of 894 participants, 544 participants did not miss work after the side-effects of the first dose. Similarly, 565 and 560 participants reported that they did not miss work after the second and third doses of the COVID-19 vaccine even if they had side-effects (Table 2).

## 4. Discussion

The rapid development and deployment of COVID-19 vaccines were crucial in mitigating the pandemic’s impact. In this study, among the 894 participants, 46.2% received an mRNA vaccine for the first dose, 45.4% received a viral vector vaccine, and a higher proportion received mRNA vaccines for subsequent doses (65.6% for the second dose and 83.6% for the third dose). This distribution reflects the increasing preference and availability of mRNA vaccines over time.

Consistent with previous studies, common vaccine side-effects included pain at the injection site, fatigue, fever, and chills [19,23,24,25]. Chrissian et al. (2022) reported that 70% of participants experienced injection site reactions, and 50% reported systemic reactions after the first dose [26]. Similarly, this study found that 54–66% of participants reported pain at the injection site across all three doses, with other frequently reported side-effects being fatigue, fever, and chills.

Although vaccine-related side-effects are generally mild and resolve within a few days, their severity can vary, sometimes necessitating medical attention and impacting work attendance [23,26,27,28]. During the pandemic, all-cause sick leave rates rose significantly compared to previous years, influenced by both COVID-19 infections and vaccine-related side-effects [29]. While some studies reported lower absenteeism among vaccinated individuals due to fewer and less persistent symptoms [30,31], others highlighted the statistically significant impact of vaccine-related side-effects on absenteeism [32].

There was no significant association between side-effects and work absenteeism after any of the three vaccine doses. Similarly, there was no significant association between the type of vaccine and missed work. This finding contrasts with some studies that reported higher absenteeism following mRNA vaccines, particularly after the second dose [33,34,35,36,37]. For instance, a survey revealed that 37% of healthcare workers experienced side-effects severe enough to necessitate missing work [33].

Regarding comorbidities, no significant association was found between underlying health conditions, side-effects, and work absenteeism. This aligns with some studies indicating that certain comorbidities, like obesity, but not others, such as hypertension or diabetes, were associated with increased systemic adverse events [23]. In this population, most participants (74.7%) did not have comorbidities, which may have influenced these findings.

Demographic factors, such as race and gender, also played a role in vaccine-related absenteeism. This study found a significant association between race and the need to miss work, with Arab participants missing work more frequently than other racial groups. This contrasts with studies indicating no association between ethnicity and work absenteeism [26]. We observed a notable association between race and the need to miss work, with Arab participants showing higher rates of absenteeism compared to other racial groups. This finding prompts further exploration into potential contributing factors.

One plausible explanation could involve cultural influences, where varying norms regarding health and illness among Arab communities might impact how individuals perceive and respond to vaccine side-effects, potentially influencing their decision to attend work. Additionally, socioeconomic factors, such as employment conditions and access to paid sick leave, could also contribute to differential absenteeism rates.

Gender differences were also notable, with males missing work more than females, despite females generally reporting more frequent and severe adverse effects [35,38,39,40].

Healthcare profession impacted absenteeism rates, with physicians needing to miss work more than nurses, contrary to findings that clinicians had lower odds of taking sick leave compared to nurses [26,28,35,37]. This discrepancy may be influenced by cultural and staffing factors unique to this study population.

However, it is important to note that this study’s sample predominantly consisted of younger individuals aged 25–34. This skewed age distribution suggests caution when extrapolating these findings to older age groups, as the vaccine’s effects and subsequent work attendance could differ significantly in older populations. Therefore, additional studies focusing on a more balanced age distribution are needed to validate these findings across different age groups.

Overall, this study underscores the importance of understanding the diverse factors influencing vaccine-related side-effects and work absenteeism. The findings highlight the need for tailored strategies to support HCWs, considering their specific demographic and professional contexts. Increasing access to paid sick leave and addressing concerns about side-effects can encourage vaccination uptake and reduce the economic burden of side-effects [41,42]. Employers should account for potential absenteeism when planning staffing to mitigate the impact of vaccine-related side-effects as vaccination efforts continue [33].

Several limitations should be noted. The sample was skewed towards younger age groups, which may affect the generalizability of the findings to older populations. Additionally, this study did not account for the interval between vaccine doses, which may influence the severity of side-effects and absenteeism. Moreover, although the sample size was substantial, a significant proportion of participants were nursing staff, which may limit the generalizability of the findings to other healthcare professions. Further research is needed to explore these factors in more diverse healthcare settings.

## 5. Conclusions

In conclusion, this research provides valuable insights into the impact of COVID-19 vaccination side-effects on work attendance among HCWs in Saudi Arabia. Through a cross-sectional-survey-based approach, we assessed the severity of side-effects following different doses of COVID-19 vaccines and their association with work absenteeism.

Our findings reveal that a significant proportion of HCWs experienced side-effects following COVID-19 vaccination, with common reactions including pain at the injection site, fatigue, fever, and chills. Despite the prevalence of these side-effects, there was no significant association between the type of vaccine administered and the need for work absenteeism. Furthermore, demographic factors such as age, gender, and healthcare profession did not significantly influence the likelihood of missing work due to vaccine-related side-effects. However, we observed variations in work absenteeism among different racial groups, with Arab participants reporting higher rates of missed work compared to other ethnicities.

This study underscores the importance of understanding the immediate practical implications of COVID-19 vaccination for HCWs. By identifying factors associated with vaccine-related side-effects and work absenteeism, healthcare organizations can better support their workforce and implement strategies to mitigate the impact on patient care and staffing.

Moving forward, future research should continue to explore the long-term effects of COVID-19 vaccination on HCWs’ well-being and job performance. Additionally, efforts to promote vaccination acceptance and accessibility among HCWs remain crucial in achieving widespread immunity and effectively combating the COVID-19 pandemic.

## Figures and Tables

**Table 1:** Participant demographics, medical history, profession, and vaccination status.

Category		Frequency	Percentage (%)
Gender	Male	146	16.3
	Female	748	83.7
Race of the Participants	African	37	4.1
	Arab	335	37.5
	Asian	503	56.3
	White	119	2.1
Location	Eastern region	60	6.7
	Middle region	576	64.4
	Northern border region	8	0.9
	North region	64	7.2
	South region	151	16.9
	Western region	35	3.9
History of Smoking	No	774	86.6
	Yes	120	13.4
History of Exercise	Every Day	74	8.3
	No	286	32
	Not every day	534	59.7
History of Comorbidity	Asthma	87	9.7
	Cardiovascular disease	5	0.6
	Cerebral disease	2	0.2
	Chronic obstructive pulmonary disease (COPD)	2	0.2
	Diabetes mellitus	20	2.2
	Hypercholesterolemia	6	0.7
	Hypertension	54	6
	Thyroid	32	3.6
	Other	18	2
	None	668	74.7
Number of Comorbidities	0	720	80.5
	1	131	14.7
	2	35	3.9
	>3	8	0.9
History of Surgical Intervention	Yes	301	33.7
	No	593	66.3
Healthcare Profession	Nurse	647	72.4
	Physician	43	4.8
	Pharmacist	10	1.1
	Technician	35	3.9
	Trainer	16	1.8
	Non-clinical	143	16
Status of Vaccination	Fully vaccinated with three dose	706	79
	Vaccinated with two doses	96	10.7
	Vaccinated with one doses	92	10.3

**Table 2:** The association between side-effects and the need for the work absence after the first, second, and third vaccine doses.

Absenteeism		Side-Effects		*p* Value
		No	Yes	
Need to be absent from work after the first dose	No	90	544	0.55
	Yes	33	227	
Need to be absent from work after the second dose	No	148	565	0.91
	Yes	33	148	
Need to be absent from work after the third dose	No	174	560	0.77
	Yes	45	115	

## Data Availability

The data generated and analyzed during the current study are available from the corresponding author on reasonable request. Please note that the link provided leads to the questionnaire used in the study: https://docs.google.com/forms/u/0/d/e/1FAIpQLSeWI57BkFxZpaltmxeXby50FHT0Ak1Lc-XDz-WuT-3XO_ajgA/formResponse (accessed on 5 June 2024).
